# Students’ learning behavior in digital education for radiation oncology

**DOI:** 10.1007/s00066-021-01858-2

**Published:** 2021-11-29

**Authors:** Hilke Vorwerk, Rita Engenhart-Cabillic

**Affiliations:** Radiotherapy and Radio-oncology, University Clinic Marburg and Giessen (Marburg facility), Baldingerstraße, 35033 Marburg, Germany

**Keywords:** Digital learning, Medical education, E-learning, Blended learning, Interactive E-book

## Abstract

**Purpose:**

Digitalization of medical education is an important trend in terms of reforming and modernizing the global education environment. It has been long requested by students and politicians. The goal of this study was to assess the student perception of a newly developed digital educational program in radiation oncology (RO) using an interactive e‑book combined with short learnings clips on a YouTube channel combined with periodic videoconferences and a forum for queries.

**Methods:**

We performed five evaluations during and at the end of two terms with multiple-choice and free-text answers. We evaluated student perception of our new digital learning scenario in three semesters: one pre-clinical and two clinical semesters. In addition, we analyzed all comments from the kMED forum, the YouTube channel, or the e‑mail contacts.

We analyzed the learning behavior of the students based on access to the videos and the number and quality of the reflective questions answered as well as the results of the final examinations.

**Results:**

The students accepted the offer for asynchronous teaching and mainly learned on weekdays (74% of the videos), but also on weekends (23%) and less on public holidays (4%). The answer quality of the reflective questions was good with over 50% correct answers on the first attempt. Learning to be on one’s own authority was very difficult for the students, even in the last clinical semesters of the medical study. Without direct intervention by the teacher, access to the learning material by the students was limited and delayed. Therefore, voluntary interim tests were performed during the first analysis term, which led to an increased number of student accesses to the videos and higher number of answers. Nevertheless, in the first analysis term, the average results in the final exam of the students who did not perform the interim test were below average at 59.1%, and the students who performed the test had better results at 69.5% but this was also not satisfactory. In the second analysis term, we taught with the same digital teaching model but with an additional scheme for learning over the term, 2‑week compulsory intermediate tests, and frequent videoconferences to answer any questions. In this term, we measured a success rate of 93% in the final exam. All annotations were very positive regarding the new educational project. The evaluations showed high acceptance of the new education program. The students stated they would prefer the new education course to be continued in future.

**Conclusion:**

Digital teaching methods make not only the type and quality of teaching transparent, but also the learning behavior of the students. Our analysis has shown that, in addition to the quality of the teaching, the clear structure and specification of the learning content per learning week as well as regular monitoring of what has been learned are of decisive importance for the learning success of the students.

**Supplementary Information:**

The online version of this article (10.1007/s00066-021-01858-2) contains supplementary material, which is available to authorized users.

## Introduction

The state-of-the-art of radiation oncology (RO) medical education in Germany in some cases starts in pre-clinical semesters, but is offered mostly in the second half of medical education during clinical semesters [1]. The predominant teaching formats for (RO) in Germany are lectures, seminars, and practical/bedside training. The main topics covered in RO teaching are general RO, radiation biology, side effects, and radiation physics. Concerning the different organ systems, the breast, prostate, head and neck, lung, brain, and gastrointestinal tumors rank the highest [1, 2]. Radiation oncology education is highly relevant to medical students to provide oncological knowledge for later residency, even non-oncological residencies. Haagedoorn and de Vries in 1998 [3] called for medical training with a focus on cancer care in general practice more than with teaching of basic science topics, detailed staging data, pharmacology of cancer drugs, and treatment protocols. They stated that the relevancy of cancer education to the reality of daily practice had evidently not been taught during medical training . Unfortunately, nothing changed in the past few decades.

Digitalization of medical education is an important trend in terms of reforming and modernizing the global education environment. In recent years, the calls from students, university panels for digitalization, and science councils to rethink and enhance digitalization in teaching have become increasingly demanding [4, 5], but the implementation is flawed in many points—there are technical, temporal, and conceptional issues that prevent progress. Digital learning is, to our knowledge, generally not implemented in RO education by default. Therefore, we developed a new digital education concept with a structured didactic curriculum for students in RO and evaluated the concept with a focus on the students’ perception [6].

Digitalization in the university environment is often ignored because of the transparency of the teaching method in quantity and quality [7–9]. However, digitalization is not a one-sided process—the learning quantity and quality of the students is also transparent. We used our new digital learning concept to analyze the learning behavior of students with the goal of adapting our program for optimal learning outcomes for the students. We should know the needs of students, providing instructions and assessing that learning has occurred in order to guide the improvements indicated [10]. Therefore, we analyzed how often and when the learning clips were played and we measured the time and the number of answers to the reflective questions in our interactive e‑book additionally.

## Material and methods

The standard curriculum for RO medical students in our university consisted of 3 weeks for basic knowledge learning, taught with pdf documents, followed by collegiate lectures for the five most important tumor entities. We developed a new interactive e‑book for collegiate training in RO consisting of a basic part for general oncology, general RO, radiation biology, and radiation physics, which cannot be omitted for the understanding of the following parts, and a clinical part with different tumor entities to replace the current curriculum.

The current possibilities of digital teaching formats for implementation of digitalization are divided into several categories: audio/video-based media (e.g., podcasts), classic digital media (e.g., digital texts), social communication tools (e.g., forums), electronic examination systems (e.g., e‑examination), and interactive tools (e.g., virtual patients; [11]). We used all of these tools for our new educational project. All of these methods have in common that they benefit greatly from a modular structure. Radiation oncology is predestined for a modular display of concise content with a clearly structured configuration. A summary of RO is possible in tabular form as shown before [12] and can be digitalized in this form directly in small sections. First of all, each tumor entity can be divided into clinical aspects and radio-oncological aspects. The clinical aspects can be subdivided into sub-sections such as epidemiology, risk factors, prognosis, etc. These appear repeatedly in the same order, and some can be omitted or added depending on the tumor entity. This results in a very clear and recurring structure that the student can learn by. The introduction of the e‑book started with a virtual 3D tour through our department. The student can virtually visit all rooms and watch short explanatory videos on the processes in radiotherapy and technology. The basic lecture was created with audio-/video-based media. New and innovative instructional video formats are short learning clips that use PowerPoint slides, whiteboards, or tablets. Initial studies were able to show that this type of educational video has a better influence on the learning effect than complex long video films [13–15]. Therefore, the learning scenario here presented uses short videos (average 3.0 min), showing an instructor explaining the material with the help of self-developed texts and graphics on a whiteboard. This was supplemented by films showing the clinical processes in an RO department. The films were shot with a camera (HDC-SD99, Panasonic) in Full HD mode. The films were uploaded using video editing software (VideoPad Professional v 8.19© NCH Software, 2020) on the YouTube platform (https://www.youtube.com/channel/UCpBHT7vSwpHdOQdVJ2IYdGA). Written consent was obtained from all persons, companies, and the medical director involved. The graphics were self-created; therefore, the copyrights belong to the creator. The consent of the participating companies to use their photos was requested and a disclaimer was placed under each video. The core of the learning scenario presented is an interactive e‑book. As a medium, e‑books contain digital content such as text, images, video, or links [16]. We built overview screens of the different pages with links to the associated short video on YouTube followed by a reflective question on the video into the e‑book. We focused on clinically relevant contents and subclassified them into basic knowledge and advanced knowledge (marked with a blue frame of the overview screens and videos).

The interactive e‑book of radiation therapy was created on the kMED learning platform (Knowledge-Based Medical Education powered by ILIAS, v5.4.10 2020-03-04) and is publicly accessible (https://kmed.uni-giessen.de/ilias/goto.php?target=lm_172102&client_id=kmed). Crosslinks to digital educational tools of other oncological departments (e.g., pathology) and crosslinks to clinical examination tutorials are implemented in the e‑book to put interdisciplinary education into practice.

## Evaluation

We evaluated three courses of two absolute digital semesters consisting of a pre-clinical semester (256 students in the fourth semester for clinical aspects in neuroanatomy teaching with interactive patient cases) and two clinical semesters (170 students in the sixth and 125 students in the tenth semester for RO). To evaluate the teaching methods used, we asked for the assessment of the interactive e‑book in comparison with other teaching methods [17]. The pre-clinical educational scenario comprised two interactive patient cases. In the first phase of the clinical education program in the sixth semester, the students had to work on the knowledge base of general oncology, general RO, radiation biology, and radiation physics. In the second phase, the students attended to the breast cancer part of the interactive e‑book. At the same time, the students of the sixth semester had to produce own short videos with a reflective question on a clinical subchapter, because passively learned knowledge can be deepened much better and saved permanently through active application [18, 19]. They had to develop videos for three other tumor entities (lung cancer, gastrointestinal cancer, head tumor), which were worked on by all students in the third phase of the semester. The clinical educational scenario in the tenth semester worked on the prostate cancer using the interactive e‑book combined with the YouTube videos. During the second term, learning progress was accompanied by an additional digital guidance with the given learning contents per week and regular videoconferences for discussion of queries.

All feedback on the kMED forum, the YouTube channel, or the e‑mail contact was analyzed in particular regarding their contents of the new educational project. One central evaluation was performed during the 5th week of the first term for all semesters and analyzed the digital success conditions, the technical aspects, the digital education, the structure, and self-organization and compared distant education with classroom teaching. The evaluation of the pre-clinical semester was accessible during the whole semester and could be performed directly after the digital course. The evaluation of the two clinical semesters was performed within the framework of the e‑exam. In the second analysis term, the evaluation took place at end of term. The evaluations analyzed the digital education, compared the interactive educational concept with PowerPoint presentations set to sound, and left room for free answers. We grouped the free answers and used the most often listed wishes to integrate these in our educational project for the following term.

## Learning behavior

We analyzed the learning behavior of two courses (5th/6th semester and 9th/10th semester) from two consecutive terms (summer term 2020 and winter term 2020/2021). The course of the 5th/6th semester consisted of 158 and 148 students and the 9th/10th semester of 125 and 132 students for the first and second analysis term, respectively. In the first analysis term, the learning scenarios were accompanied by a forum for communication to the lecturer and finished by an e‑exam. During the first phase of the education program in the 5th/6th Semester, the students had to work on the basic knowledge of oncology, RO, radiation biology, and radiation physics. In the second phase, the students attended to the breast cancer part of the interactive e‑book. At the same time the students had to produce their own short video in small groups including a reflective question to a clinical subchapter. The reason for this procedure was that passively learned knowledge can be deepened much better and saved permanently through active application [18, 19]. All together they had to develop videos for three other tumor entities (lung cancer, gastrointestinal cancer, head tumor), which were worked on by all students in the third phase of the semester. Because of our interim analysis of the learning behavior of the students, we provided an optional digital pre-test 2 weeks during this phase before the e‑exam at end of term. We analyzed the accesses of the students to the interactive e‑book and the reflective questions as well as to the videos on the YouTube channel. We analyzed the daily and weekly access for the following sections: general medicine, general oncology, general RO, radiation biology, radiation physics, breast cancer, lung cancer, rectal cancer, and glioma. We correlated the access of the students with the phase of the semester. The 9th/10th semester had to work on prostate cancer. For the same reason as for the 5th/6th semester, we provided an optional digital pre-test 2 weeks before the e‑exam at end of the semester and analyzed the daily and weekly access to the section on prostate cancer of the interactive e‑book and the YouTube channel videos.

Based on our analysis of the first analysis semester, we established frequent interim tests for the second analysis term for both semesters. We analyzed only the number of answers in the interactive e‑book because of the assumed bias in the YouTube videos. The number of accesses on the YouTube channel has the bias of possible external accesses. The YouTube channel was newly implemented at the beginning of the first analysis term (first videos was uploaded 3 weeks before beginning of the term) and had been accessed 1209 times before the term and 30,000 times at end of term. Therefore, we assumed that during this term most of the accesses are from the students of our department. At the beginning of the second analysis term, the video channel had over 500 followers, so that the number of video accesses could no longer be used for evaluation. The interactive e‑book analyzed was only accessible for the students of our department.

## Results

All annotations in the kMED forum, the YouTube channel, or the e‑mail contact were very positive regarding the new educational concept and some asked for more content (see Appendix).

### Results of the central evaluation

In total, 24 students completed the central evaluation questionnaire (15 female, 9 male, age: 22.6 ± 2.5). The digital success conditions were completely fulfilled with our new digital educational program. They were evaluated with an average score of 4.4 ± 0.3 on a scale from 1 to 5 points. The student can learn temporally and spatially independent (5.0 points) and decides herself/himself, what she/he wants to learn (4.0 points). The trainable contents were elucidated in the introduction of the interactive e‑book (4.5 points) and the necessary materials (e.g., scientific papers besides the interactive e‑book contents) were provided (4.4 points). The learning results could be deliberated with the reflective questions in the interactive e‑book (4.6 points). Overall, participants were encouraged through the educational scenario to contribute actively one’s services (4.4 points) and deal with the contents frequently (4.2 points). The technical implementation of the new educational program of RO was rated as felicitous (4.7 points). No personal groups were favored or disadvantaged (4.4 points). There were no technical problems (1.2 points for technical problems).

In the part on structure and self-organization of the questionnaire, the students could choose between “would be very helpful” (5) to “would be not helpful” (1). The students opted for no peer-review (2.1) but instead for more feedback by the lecturer (3.5) and for more contact with the lecturer (3.3). The students rated the information provided in the tools used as adequate (2.9). They stated that a more restrictive regulation of the course would not be helpful (2.1), but that digital schedules (3.9) and interim tests (3.5) could be helpful.

Overall, 73.65% of the students wished for a content of digital education in the RO course in future and a large part with asynchrony education (86.63%). When students were asked if they could rewind time to the beginning of the semester and could choose between a regular classroom course and a digital course what their choice would be was answered as classroom teaching by 31.5% of the students and digital education by 68.4% of the students.

In the free-text evaluation, the students stated that they would prefer the education course to be continued in future.

### Results of the pre-clinical evaluation (first term)

In total, 256 students took part in the two interactive patient cases and ten students completed the evaluation questionnaire (5 female and 5 male). The total digital pre-clinical course was rated with a school grade of 1.6 ± 0.7. The crosslinked external clinical examination videos, the five short videos on the YouTube channel, and the pictures used received good assessments (1.7 ± 0.5 and 1.7 ± 0.6). The students wrote in the free-text evaluation that this new concept is interesting and attractive. The interactive pictures were debated as being very good to not comprehensible.

### Results of the clinical evaluation (first term)

In total, 125 students worked on the section on prostate cancer in the interactive e‑book and 122 students completed the evaluation questionnaire. The students assessed the interactive e‑book in combination with the YouTube videos as very suitable for teaching clinical aspects (1.67 ± 1.01). They rated the digital overviews as very helpful for learning (1.71 ± 1.02).

Overall, 147 students completed the basic chapter and the chapters for breast cancer, lung cancer, rectal cancer, and brain tumors in the interactive e‑book and 144 students completed the evaluation questionnaire. The students assessed the interactive e‑book in combination with the YouTube videos as suitable for teaching clinical aspects (2.32 ± 1.10). They rated the digital overviews as helpful for learning (2.19 ± 1.15).

The students compared the leaning scenario with the interactive e‑Book with PowerPoint presentations set to sound, and 61.3% rated the new learning scenario as more or much more suitable, 28.2% as comparable, and 10.6% as less suitable.

All free-text evaluations were very positive toward the new learning scenario. On student summarized the opinions: “The lecturer has shown with how much effort and love digital education can be performed. The combination between YouTube channel and interactive e‑book is perfect, because they are complementary. The YouTube channel is well arranged and with the playlist the student can selectively learn what he or she needs at that moment. The lecturer managed to put it all in a nutshell with 2–5-min videos. In comparison with audio lectures, parts can be skipped, repeated, or learned at the speed of the individual student. During a 90-min lecture, the students can lose the overview very easily. Compared with other kMED learning courses, the RO e‑book is clearly arranged and interactive, combined with questions and linked to the associated video if one answered the question wrong. With the forum there was always communication with the lecturer for any questions. This format is not only reasonable for students but also for apprentices, patients, and interested layman.” The students remarked on many ideas for suggestions, which are answered in the discussion in detail. All comments are attached in a supplementary file.

### Results of the clinical evaluation (second term)

A total of 19 students completed the evaluation questionnaire. The conditions for digital success were completely met with our new digital educational program. They were evaluated with an average score of 4.4 ± 0.8 on a scale from 1 to 5 points. The student can learn temporally and spatially independently (5.0 ± 0.0 points). Overall, participants were encouraged in the educational scenario, besides the reflective questions and short videos, to deal with the contents frequently (3.9 ± 0.7 points). The technical implementation of the new educational program of RO was rated as felicitous (4.8 ± 0.3 points).

In the part for structure and self-organization of the questionnaire, the students could choose between “would be very helpful” (5) to “would not be helpful” (1). The students stated that considerable contact with the lecturer would be helpful (4.1). The students rated the interim as helpful (4.3). During this term, we performed an additional learning guide through the term. Afterward the students stated that additional guidance would be neither more nor less helpful (3.0), but the actual guidance was helpful (4.0). When students were asked if they could rewind time to the beginning of the semester and could choose between a regular classroom course and a digital course what their choice would be was answered as classroom teaching by 55.5% of the students and digital education by 44.4% of the students.

## Relation between video accesses and reflective questions answered

At the beginning of the term, the relation between video accesses on the YouTube channel and reflective questions answered in the interactive e‑book after 1 week was 11.7 more video accesses than answered questions and decreased rapidly to 3.99 after 2 weeks. From that point, the relation decreased almost linearly to 2.8 at the end of term, which means about three times more video accesses than reflective questions answered.

## Independent asynchronous learning

We counted the number of digital accesses to the short videos on the YouTube channel and the reflective questions on weekdays, weekends, and public holidays during the first analysis term to analyze the asynchronous learning behavior of the students. During weekdays we measured on average 490.0 accesses (74% of all accesses) to the videos per day, during weekends 371.7 accesses (23% of all accesses) per day, and on public holidays 287.4 accesses (4% of all accesses) per day with 4 days of public holiday during the summer term. The number of completed answers to the reflective questions of the interactive e‑book on weekdays were 159.1 per day (67% of the answers), on weekends 119.5 per day (31% of the answers), and on public holidays 69.3 per day (2% of the answers).

A subanalysis on the weekdays from Monday to Thursday showed 518.5 accesses to the videos and only 365.7 on Fridays, which was similar to the number of accesses on weekends. The number of answered questions was 167.7 per day from Monday to Thursday and 141.2 on Fridays, some more than on weekends.

## Quality of answers to the reflective questions from the interactive e-book

**Fig. 1:**
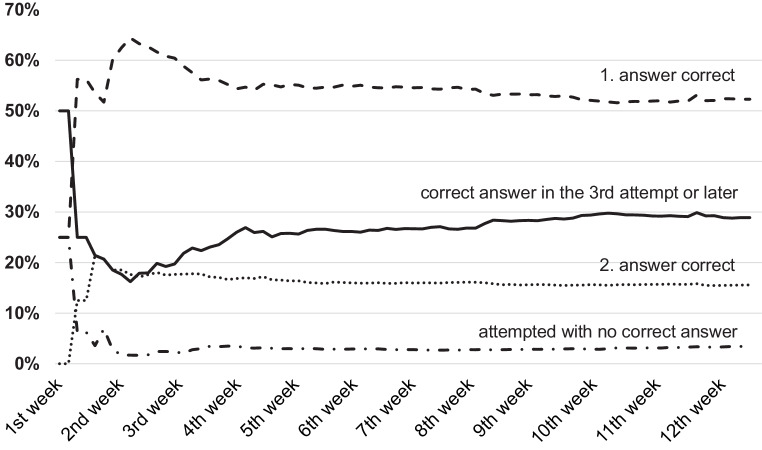
Response style to the reflective questions of the interactive e‑book during the first analysis term. *Dashed line* correct answer in the first attempt, *dotted line* correct answer in the second attempt, *solid line* correct answer in the third or later attempt, *dashed and dotted line* student attempts to answer the question but did not get the correct answer

During the 2nd week of the first analysis term after the 1st week with testing, the students answered 62% of the reflective question in the first attempt, 18% in the second attempt, and 18% in the third or later attempts, and 2% of the questions were processed but not answered (Fig. 1). On average the students needed 1.61 attempts for the right answer. The percentage of questions answered in the first or second try decreased over the weeks to 52% and 16%, respectively, whereas the questions answered during the third try or later increased up to 29%. The average needed for the right answer increased up to 1.84 tries.

We found the same rate in the second analysis term with 50% answers in the first try, 18% in the second try, 29% in the third try or later, and 3% of processed but not finished questions from the 2nd week up to the end of term. The average needed for the right answer was 1.85 tries.

## Learning behavior in the 9th/10th semester

**Fig. 2:**
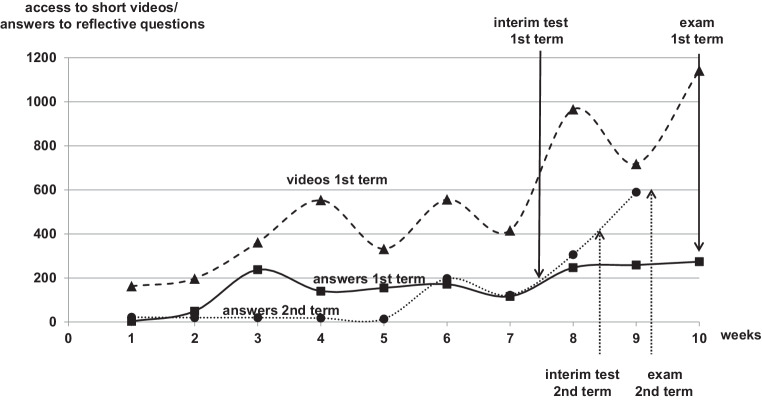
Accesses to the short videos on the YouTube channel and number of reflective questions answered on prostate cancer per week (9th/10th semester). *Dashed line with triangles* accesses to the videos during the first analysis term, *solid line with squares* answers to the reflective questions during the first analysis term, *dotted line with circles* answers to the reflective questions during the second analysis term. Voluntary interim test between 7th and 8th week during the first analysis term; obligatory interim test between 8th and 9th week during the second analysis term; obligatory e‑exam in week 10 during both analysis terms

During the first 2 weeks of the first analysis term, the students of the 9th/10th semester began to work on prostate cancer and we measured 261.5 ± 98.5 accesses to the prostate cancer videos during the first analysis term (Fig. 2); each student answered on average 23 of the reflective questions of the interactive e‑book. We found in the 3–7th week an average of 444.2 ± 94.8 video accesses per week and the students answered on average 164.2 ± 41.2 of the prostate cancer reflective questions per week. At the end of the 7th week, we found 2581 accesses to the 33 prostate cancer videos, which corresponds to 20.8 accesses per student (62.6%). Overall, 873 questions were answered during the first 7 weeks, which corresponded to 7.0 questions of the 23 prostate cancer questions answered per student during the first two phases (30.6% of the question). A total of 62 of the 125 students finished the optional interim test. After the optional interim test, the number of video accesses increased to an average of 917.0 ± 146.6 and the questions answered to 260.0 ± 11.0 (8th–10th week). Before the end examination, 5332 video accesses were recorded, which corresponds to 42.7 videos per student (129.3%). The number of questions answered was 1653, which corresponds to 13.2 questions per student (57.5% ± 7.0% with a minimum of 50.4% and a maximum of 72.8% per question).

For the second analysis term, we planned the learning phase for the 6th–8th weeks followed directly by an obligatory interim test. Some students had already answered several questions during the first 5 weeks and we found an average of 60.0 ± 25.8 questions answered per week. During the 3 weeks, which were planned for learning (weeks 6–9), we found an average of 208.7 ± 86.0 answers per week. The e‑exam was planned on the 10th week. During the 2 weeks between the interim test and the e‑exam, we found another unexpected increase from 590 questions answered in week 9.

In the e‑exam at end of term, the students achieved on average 54.1 ± 28.6% of the points. In the subanalysis of the students who completed the voluntary interim test, the average result was 66.5 ± 25.2% in comparison with the students who did not complete the test with only 50.8 ± 20.8% of the achievable points. In the e‑exam during the term with the obligatory interim test, the students achieved 97.9% of the points.

## Learning behavior in the 5th/6th semester

The schedule for the 6th semester consisted of three phases. The first phase (weeks 1–3) comprised the acquisition of basic knowledge. In the second phase (weeks 4–6), the students had to learn about breast cancer and had to produce their own short video simultaneously, which had to be submitted at the end of this phase. In the third phase (weeks 7–13), the students had to learn about the three tumor entities: lung cancer, glioma, and rectal cancer.

**Fig. 3:**
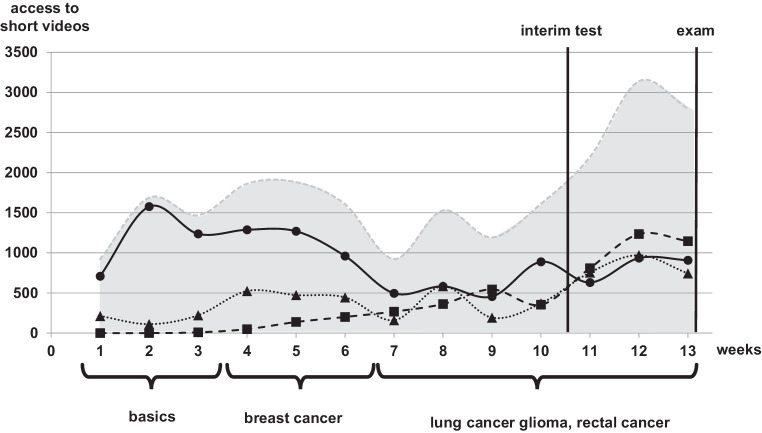
Accesses to the short videos on the YouTube channel per week during the first analysis term (5th/6th semester). *Gray area* number of all accesses, *solid line with circles* basic knowledge, *dotted line with triangles* breast cancer, *dashed line with squares* lung cancer, glioma, and rectal cancer

**Fig. 4:**
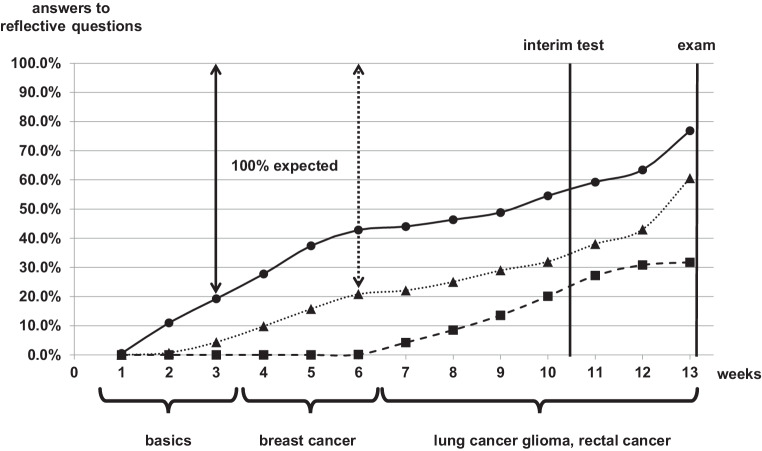
Relative number of reflective questions answered per week during the first analysis term (5th/6th semester). *Solid line with circles* basic knowledge, *dotted line with triangles* breast cancer, *dashed line with squares* lung cancer, glioma, and rectal cancer

During the first 3 weeks, the students worked on the basic knowledge (Fig. 3). We found an average of 1174.3 ± 357.1 video accesses per week, which corresponds to 20.7 accesses per student for the 42 basic videos in the first phase. After the 3rd week, the students had answered 1064 of the basic reflective questions in the interactive e‑book, which corresponds to 6.7 questions answered of the 37 basic questions per student (Fig. 4). The students had watched 53.1% of the basic videos and answered 19.2% of the basic questions during the first phase.

The second phase was planned for learning about breast cancer. We found an average of 481.0 ± 32.6 video accesses per week, which corresponds to 3.0 accesses per student for the 33 breast cancer videos in the second phase. After the 6th week the students had answered 757 of the breast cancer reflective questions in the interactive e‑book, which corresponds to 4.8 questions answered of the 23 basic questions per student. However, some students had already completed these tasks during the first phase of the term. Thus, the students had watched 38.1% of the breast cancer videos and answered 20.8% of the breast cancer questions after the second phase.

The third phase was planned for learning about lung cancer, glioma, and rectal cancer. During weeks 7–10 we found an average of 382.0 ± 202.0 video accesses per week, which corresponds to 2.4 accesses per student for the 66 short videos of the third phase per week. After the 10th week, the students had answered 2986 of the reflective questions of the third phase in the interactive e‑book, which corresponds to 4.8 questions answered of the 94 basic questions per student.

The students also worked on the basic and breast cancer videos and questions during the third phase. Hence, we found 29.8% questions answered for all the categories together. In summary, this was not enough time for 3 weeks prior to the exam. Therefore, we executed a voluntary interim test for the students after the 10th week. Afterwards the number of accesses to the videos increased from 382 per week to 1063 ± 183.2 per week and the number of questions answered increased from 4.2% answers per week during weeks 7–10 to 5.6% during weeks 11–13.

Nevertheless, the number of total reflective questions answered at end of term was only 46.5% with 76.9% basic answers, 67.7% breast cancer answers, and 31.7% answers to the questions of the third phase.

During the second analysis term, the number of questions answered increased from 381 in the 1st week to 2603 during the 3rd week, directly before the first interim test. During the next 4 weeks the number of questions answered was nearly constant at 1402.0 ± 306.2 answers per week. After 7 weeks, every student had answered on average 55.7 questions. During the next 2 weeks, which were planned for breast cancer knowledge, the students only answered 440 ± 84.7 questions. This increased during the next 2 weeks, planned for learning about glioma, up again to 868.5 ± 134.5 answers per week. The number of total reflective questions answered at end of term was only 52.5%, not much more than after the first term.

In the e‑exam at end of term, the students achieved on average 72.0 ± 15.1% of the points. In the subanalysis of the students who executed the voluntary interim test, the average result was 73.1 ± 14.0% in comparison with the students who did not execute the test, with only 68.7 ± 14.5% of the achievable points. In the e‑exam from the term with the obligatory interim test the students achieved 87.4 ± 8.1% of the points.

## Discussion

Social networks are the basis of communication among the younger generation and medical education should make maximum use of them in order to optimally reach this generation and thus maximize learning success. However, it should be clear that growing up with digital media does not necessarily lead to a meaningful use of this media in the context of learning [20]. A study published by the University Forum Digitalization in 2016 showed great benefit for the learning process only if the digital media were an integral part of the university teaching [21]. Currently, medical studies mainly use electronic examination systems, subject-specific databases, videos, and social networks. Only 23% of medical students use all types of media and 47% use only online exams in a passive, consuming role as digital learning methods. This means that the students spend a lot of time on social networks and we can use this factor to convey meaningful content. It is a great opportunity, especially for radiotherapy as a small cross-sectional medical field, to raise awareness in our field and highlight its possibilities. In addition, we can use the limited time for the attendance classes in RO to familiarize students with the field of RO. Some students stated in the free text of the evaluations that they had no special interest in RO before the course but asked for clinical training after the digital course.

## Evaluation

Medical faculties have very different kinds of curricula, some have seminars or lectures and others have extensive curricula with representation of RO in all clinical semesters [1]. In our faculty, RO is represented in nearly all clinic semesters, but in only three semesters do we have our own seminars: the 4th semester (preclinical) and the 5th/6th and 9th/10th semester. We developed a new digital education format for these three semesters and analyzed the success with four different evaluations. Teaching students in medical education programs is a big responsibility and we have to know the requirements of the students, assess the students’ learning, and improve our medical education [10]. Therefore, the evaluation of medical students is an important formative and summative assessment method as used in this analysis [22]. The integration of RO in preclinical education has been commented on [23]. Our preclinic evaluation showed a very good rating of our new digital patient cases. The interactive pictures were not widely understood, so that we reworked them for the next semester. Our main RO lectures are settled in the 5th/6th and 9th/10th semester. The clinical evaluation showed general satisfaction of the students with the learning scenario consisting of the YouTube videos combined with overview screens and reflective questions in the interactive e‑book. The central evaluation during the fifth semester week analyzed the digital success conditions, the technical aspects, the digital education, the structure and self-organization, and compared distant education with classroom teaching. The results showed very good acceptance of the new educational program by the students, who want the work with the interactive e‑book to be continued with additional practical lessons. We are planning to combine the interactive e‑book with practical lessons with construction of a thermoplastic mask, realized by the students, conducting a CT scan of a phantom and letting the students define the target volume on a prepared patient case, and treating the phantom at the linac.

Digitalization has led to time-independent learning scenarios for students, who can freely decide when and where they want to learn. This was possible with the new education program for RO as rated by the students. However, this must always be assessed with the help of milestones, such as interim examinations etc., so that a planned learning success is achieved within a predetermined period of time, which is demanded by the students. Not only in the central evaluation (interim tests could be helpful) but also in the clinical evaluation, students asked for interim tests, which were performed biweekly in the following term for each tumor entity. The student can sit the examination digitally at home for 1 h. Pass the interim tests is currently the entry restriction for the final examination. Some students wished for interactive conferences to ask questions about the learning contents. Currently, we offer biweekly question sessions 1–2 days before the biweekly interim tests. Several students asked for overview screens and reflective questions, which revealed that the students had not found the link to the interactive e‑book. Therefore, and because other students demanded for a better breakdown of the course structure in the clinical evaluation and stated that a time schedule could be helpful in the next semester, we provide detailed guidance through the interactive e‑book and the whole term with detailed information on the course. We additionally created a picture of the time schedule as an overview with milestones and the time of the question rounds and interim tests. Additionally, pictures and a detailed schedule for the whole semester was provided for the second term, as requested by the students. Some students asked for lecture notes containing the overview screens, which are to be provided at the end of the interactive e‑book in future. A written script contradicts the idea of the learning scenario and only supports short learning phases before the e‑exam and will not be created.

The flexible use of time by the teacher themselves is often overlooked but is just as important as it is for the student. When using forums instead of chats the teacher can also decide when and where to communicate with the students. The forums also offer students and lecturers the opportunity to communicate fast and efficiently with the entire group. The use of a forum as a communication tool in the clinical semesters was appreciated by the students.

The students requested no peer-review but instead more feedback by the lecturer and more contact with the lecturer. It should be mentioned that the students had the possibility to make an anonymous peer-review of the student-made short videos, but no peer-reviews were delivered. And this questionnaire was carried out 1 week before the deadline for the collegiate videos, at which time point all students got two individual feedback reports, one for the short video and one for the reflective question. Feedback has been shown to be effective in improving student skills [21]. In the next semester, in which only digital education is possible, more information about the upcoming feedback will be provided in the introduction chapter. The introduction will be a short video and not only a text document to explain in detail the organization of the education tool.

The widely used PowerPoint presentations set to sound are a very good beginner’s tool for digitalization, but not a digital educational program. Therefore, we asked the students at the end of the term, with PowerPoint presentations generally used in nearly all medical departments, to state their opinion of these presentations compared with the interactive e‑book concept. Overall, 61% of the students rated the new interactive e‑book concept as more suitable than the concept with PowerPoint presentations and only 10% preferred the PowerPoint presentations.

## Learning behavior

There has been long ongoing discussion of whether online learning or face-to-face is teaching is better [24–29]. Mostly the effectiveness or the performance of the courses was analyzed. Often the students were asked for their evaluation of the course. To our knowledge, the learning behavior of the students was not analyzed in detail. We can provide the best online learning courses, but they will be useless without effective learning behavior on the part of the students. Therefore, we analyzed when and how the students used our interactive e‑book, to provide the optimal support for the best learning outcome and student success in the future.

The YouTube channel of our leaning program was initiated at the beginning of the first term and therefore was not familiar to other external users than the students. The close relation between the video accesses and number of questions answered suggests that most accesses during the first term were student related. The number of followers increased over time, and therefore we did not analyze the video accesses in the second term due to the high number of external accesses. Even though the interactive e‑book is publicly available, the analysis of the answers to the questions of the interactive e‑book is directly correlated with the tutored students and thus showed no bias of external users. At the beginning of the semesters, approximately 10 more accesses to the videos were measured than questions answered. After 2 weeks, the students began to answer the reflective questions consistently and we found four video accesses per question answered. During the term, the relationship decreased linearly to 2.8 video accesses per question answered. This is emphasized through the young age of the YouTube channel, which was initiated only 3 weeks before the start of the first analysis term. Therefore, we assume that the students began the semester by watching the videos followed by answering the questions combined with repeating some videos and watching new ones. We know from our evaluations that some students watched the short video several times, especially directly before the e‑exam. In summary, even under consideration of the external video accesses we can state that the students watched two to three times more videos than answering questions.

Independent asynchronous learning scenarios are becoming increasingly important and demanded by the students [4, 5]. E‑learning technologies offer learners control over content, learning sequence, pace of learning, time, and media, allowing them to tailor their experiences to meet their personal learning objectives [30]. We demonstrated that the students used our offer to learn on any day of the week. The students preferably watched the videos and answered the reflective questions of the interactive e‑book on weekdays (74%/67%), less on weekends (23%/31%), and mostly not on public holidays (5%/2%). Furthermore, we showed that the students learned to an increasing degree on Monday to Thursday and much less so on Fridays. As assumed, most work was done during weekdays but nearly one-third was done during public holiday and weekends. An independent asynchronous learning is not only possible with our new learning scenario but is already embraced by the students.

The students needed an average of 1.84 tries in the first analysis term and 1.85 tries in the second analysis term to find the correct answer to a reflective question (Fig. 1), which indicates a good balance in difficulty of the developed questions. At the beginning of the semester, nearly two thirds of the students answered the reflective questions correctly in the first try, one-fifth in the second, and one-fifth in a later try. At the end of the semester, only half of the questions were answered in the first try and one-third in the third or later try. A likely explanation could be that the better students worked with the interactive e‑book at the beginning of the semester and finished the work early. Another explanation could be that in the time before the end of the semester and the e‑exam, more students just attempted the questions until they found the right answer.

## Learning behavior in the 9th/10th semester

Asynchronous learning methods are demanded by the students. Our first attempt was an absolute free and asynchronous learning offer with digitalization of all material and no stopover. Already at the beginning of the first analysis term, we observed much fewer accesses to the learning videos and much fewer answers to the reflective questions than expected (Fig. 2). At our university, RO is a small interdisciplinary profession with only one course in the 9th/10th semester. Therefore, we suppose that most of the students did not start with the contents of the prostate cancer section before the 3rd week of the semester. During the 3rd–7th weeks, the average number of video accesses was 164.2 per week, which corresponds to 1.3 videos per student. The number of all accesses to the prostate cancer videos under neglect of the number of external video accesses was 2581 in the first 7 weeks, which corresponds to 20.6 videos per student from a total of 33 prostate cancer videos (62.6%). And with consideration of external accesses the number of performed learning videos by the students could only be lesser than measured. At this time only 30.1% of the expected answers were completed by the students, which directly correlate to the learning progress of the students. Therefore, we developed an optional digital interim test at this time point 2 weeks prior to the e‑exam as a “gentle reminder” for learning.

Afterwards the average number of video accesses increased from 444.2 to 942.0 per week and the number of questions answered increased from 164.2 to 260.0. We assume that providing the interim test changed the learning behavior of the students. The number of video accesses during the whole term was 5407.0, which corresponds to a maximum of 42.9 accesses per student to 33 videos considering the unknown number of external accesses. The assumption suggests that all students had seen the videos at least once, but the number of questions answered correlated to the students showed another result with only 57.0% of questions finished. All questions had been answered by a minimum of 50% of the students and no question by more than 72.8% of the students; 27.2% of the students did not answer any question. This was reflected in the outcome of the e‑exam at end of term. The interim test had been completed by half of the students, who achieved better results in the e‑exam than the students who skipped the test. We believe that the same students who answered the reflective questions completed the interim test and achieved better results in the e‑exam.

The e‑exam on RO in our university is combined with the e‑exam of diagnostic radiology and nuclear medicine, who used predominantly time-tested questions. However, RO represents only 24% of the e‑exam, and therefore it is possible for the students to skip learning RO and obtain the certificate anyway. The part of RO would have been passed by only half of the students. The total e‑exam was passed by 120 of the 125 students. Another explanation could be that in the progress of the digitalization of the course, we changed the subject of the teaching in the 9th/10th semester from head and neck cancer to prostate cancer, and the students simply learned the questions from the previous terms. The digital education program led not only to more visibility of the teaching behavior but also of the learning behavior. We were surprised by these finding regarding learning behavior, which was expected for scholars but not for medical students in a clinical semester. Our opinion at that time was that RO should be taught without the interference of diagnostic professions and a more restrictive regime is needed to motivate all students to learn RO and achieve better results.

During the second analysis term we found about the same number of questions answered during the first 5 weeks. During the planned learning phase (weeks 6–8) we found an increasing number of answers, up to 720 questions answered in week 8 directly before the compulsory interim test. We did not expect an additional increase in answers after the test. However, after the test, the number of questions answered showed the highest value in week 9 with 590 answers per week. We suppose the students realized during the interim test that they needed more learning or more questions answered for the e‑exam. In both terms, the learning intensity of the students grew after the voluntary or obligatory interim test. In this term with obligatory interim tests, the students achieved 97.9% in the RO part of the e‑exam and 100% of the students passed this part. During both terms, we used only newly developed questions that could not be simply recited by the students. Therefore, we consider that the compulsory interim test was the reason for this much better result.

## Learning behavior in the 5th/6th semester

After our experience with the 9th/10th Semester, we analyzed the learning behavior of the students in the 5th/6th semester and found similar results. These students were taught about the oncological and radio-oncological basics and many different tumor entities, which involves much more learning effort than in the 9th/10th semester. In the first analysis term after the first phase (weeks 1–3), the students were expected to have obtained the basic knowledge, but only 19% of the questions were answered (Fig. 2). After the second phase (weeks 4–6), only 20.8% of the breast cancer question were answered. In parallel, the number of basic questions answered increased to 41.7% at the end of the second phase, which was not intended for this period. In the middle of the third phase (after week 10), 55% of the basic questions, 32% of the breast cancer, questions and on average 0.1% of the other tumor entity questions (lung cancer, glioma, and rectal cancer) were answered. Analogous to the 9th/10th Semester, we offered a voluntary interim test with the consequence that the average percentage of answered questions increased from 3% per week to almost double at 5.6% per week. However, regardless of this effort, directly before the e‑exam only 46.5% of all questions were answered by the students.

The learning behavior during the second analysis term was as expected, except for the 8th and 9th week. During the 8th and 9th week, the students were expected to have worked on the breast cancer part. We can only guess that the students had no need to answer the questions because their understanding of the videos was good enough without the questions, or because the results of this part of the interim test and the e‑exam was as good as the other parts of the RO course. The students worked with the expected parts of the interactive e‑book. The rate of questions answered, at only 52.5% after the whole term, was again much lower than expected. Again, we can only suppose that the short videos were mostly sufficient for learning by oneself. On the other hand, 12,510 questions were answered by the students during the second analysis term, which indicates the importance for the students of these optional reflective questions.

The students who did not complete any interim test (during the first analysis term), achieved on average 69% of the points in the e‑exam, and the students who completed the voluntary interim test attained 73%. In the following analysis term with the compulsory interim test, the students achieved 87% in the RO part of the e‑exam and 100% of the students passed the RO part of the exam. During these terms, we only used newly developed questions, which could not simply be recited by the students. Therefore, we believe the compulsory interim test was the reason for this much better result, in agreement with the analysis from the 10th semester. These results demonstrate that besides the quality of the learning method, regular interim tests are fundamental for the learning outcome.

The assessment of the interim tests by the students was much the same. In our evaluation of the first analysis term, the students rated interim test as a bit helpful (3.5 of 5.0 points) and in the second analysis term as very helpful (4.3 of 5.0 points). Surprisingly, the students appreciated our interim tests. During the second analysis term, 47% of the students reached the necessary point after three of the five interim tests and 99.3% after four of the five interim tests to get access to the e‑exam. Regardless, 96% of the students wrote the fourth interim test and 91.7% the fifth interim test, even though this was no longer mandatory for many students.

## Summary

The learning scenario with an interactive e‑book combined with short videos on a YouTube channel is feasible and rated highly by the students. After the first term, 68.4% of the students required digital asynchronous teaching to be continued, which decreased to 44.4% after the second digital term. After two terms of digital-only teaching, the students were tired of only digital teaching and wished for more presence not only via videoconferences but more so in face-to-face teaching with more room for asking questions than in a video conference. Our opinion is that the best digital system can only accompany but never replace face-to-face teaching lessons. Therefore, we plan to continue our teaching project with its blended learning concepts, combining teaching the basics digitally and achieving more room for practical exercises. Digital teaching methods make everything transparent—the quantity and quality of teaching, but also the quantity and quality of learning. The analysis of this is feared by both sides, but it provides plenty of room for new inventions. We know that the use of digital teaching methods poses a great challenge to the teacher, but with our analysis we demonstrated that the challenges for the students are a good deal bigger. The loss of guidance through the term is a widely underestimated problem. Beside the quality of the teaching, a structured sequence of the learning units with regular targets are the main requirement for a good learning results.

## Supplementary Information

Central questionnaire, Pre-clinical questionnaire, Clinical questionnaire, Free text evaluation of the central questionnaire, Free text evaluation of the pre-clinical questionnaire, Free text evaluation of the clinical questionnaire
