# Supplementary material for: Students’ learning behavior in digital education for radiation oncology
Source: Strahlenther Onkol. 2021 Nov 29;198(1):12–24. doi: 10.1007/s00066-021-01858-2 (PMC8760198; doi:10.1007/s00066-021-01858-2)
Supplement: Supplementary file 1 — Central questionnaire, Pre-clinical questionnaire, Clinical questionnaire, Free text evaluation of the central questionnaire, Free text evaluation of the pre-clinical questionnaire, Free text evaluation of the clinical questionnaire [file 66_2021_1858_MOESM1_ESM.docx]

# Supplement

# Central questionnaire

**I. Digitale Gelingensbedingungen**

1. In der Veranstaltung wird der mögliche Nutzen der Inhalte thematisiert.
2. In der Veranstaltung kann ich selber entscheiden, wie ich lernen möchte.
3. In der Veranstaltung wird alles bereitgestellt, sodass ich effizient lernen kann (z.B. Materialien, Technik).
4. In der Veranstaltung habe ich regelmäßig Gelegenheiten, meinen Lernfortschritt und meine Lernergebnisse zu reflektieren
5. Für die Veranstaltung kann ich zeitlich flexibel lernen (z.B. mal morgens, mal abends)
6. Insgesamt regt die Veranstaltung dazu an, aktiv mitzuarbeiten.
7. Die Veranstaltung motiviert mich, mich regelmäßig während des Semesters mit den Inhalten auseinanderzusetzen.
8. Die Interaktion und Kommunikation wird durch die in der Veranstaltung eingesetzten Lernformate und Werkzeuge …

**II. Technische Aspekte**

1. Wie zufrieden sind Sie insgesamt mit der technischen Umsetzung der digitalen Lehre in dieser Veranstaltung?
2. Das in der Lehrveranstaltung eingesetzte digitale Element scheint keine Personengruppe zu bevorzugen oder zu benachteiligen.
3. In dieser Veranstaltung war das Ausmaß technischer Probleme (z.B. Tonprobleme, instabiles Netzt etc.) bislang …

**III. Digitale Lehre**

1. Der Anteil asynchroner Lehre (d.h. hochgeladenen Inhalte für das Selbststudium, die zeit- und raumunabhängig genutzt werden können) in dieser Veranstaltung liegt bei …
2. Zukünftig sollte der Anteil asynchroner Lehre in dieser Veranstaltung bei … liegen.
3. Wie hoch sollte der Anteil an digitaler Lehre in dieser Veranstaltung zukünftig (für die Zeit nach Corona) sein?

**IV.Struktur und Selbstorganisation**

1. Digitales lernen erfordert viel Selbstorganisation von den Studierenden. Welche der folgenden Maßnahmen würden Studierenden helfen, das Arbeiten für diese Veranstaltung besser zu strukturieren?
   1. Mehr Kontakt zu Dozierenden
   2. Peer-Feedback (z.B. durch gegenseitige Beurteilung)
   3. Zwischentest zur Überprüfung des eigenen Wissenstandes
   4. Restriktivere Steuerung (z.B. durch ILIAS)
   5. Zeitpläne zur Verfügung stellen
   6. Mehr Informationen zu verwendeten Tools zur Verfügung stellen
   7. Mehr Feedback durch Dozierende zu erarbeiteten Inhalten

**V.Distanzlehre vs. Präsenzlehre**

1. Wenn Sie die Zeit zurückdrehen könnten und es wäre nochmal Semesterbeginn: Würden Sie diese Veranstaltung lieber so wie jetzt oder lieber in regulärer Präsenzlehre besuchen?

# Pre-clinical questionnaire

1. Bitte bewerten Sie das generelle Lernmodul mit Schulnoten 1- 6 (sehr gut bis ungenügend)
2. Bitte bewerten Sie die Auswahl der Kurzvideos mit Schulnoten 1- 6 (sehr gut bis ungenügend)
3. Bitte bewerten Sie die verwendeten Bilder (interaktive Bidler, MRT-Bilder etc.) mit Schulnoten 1- 6 (sehr gut bis ungenügend)
4. Bitte geben Sie hier im Freitext Ihre Beurteilung, Ideen und Verbesserungsvorschläge an:

# Clinical questionnaire

1. Wie gut ist das Konzept des "Interaktiven E-Books der Radioonkologie" mit den  Kurzvideos auf YouTube geeignet, um das Wissen bezüglich des Prostatakarzinoms auf digitalem Weg zu vermitteln? Bitte bewerten Sie mit einer Schulnote 1- 6 (sehr gut bis ungenügend).
2. Wie hilfreich waren die Tafelbilder für einen schnellen Überblick und das Lernen für die Klausur? Bitte bewerten Sie mit einer Schulnote 1- 6 (sehr gut bis ungenügend).
3. Wie beurteilen Sie das Konzept des Interaktiven E-Books mit den Kurzvideos und den Tafelbildern bezüglich der Wissensvermittlung im Vergleich zu besprochenen Powerpointpräsentationen?
4. Hier können Sie im Freitext Ideen, Verbesserungsvorschläge und Beurteilungen zum Konzept in der Strahlentherapie angeben.

# Free text evaluation of the central questionnaire

- Es könnte noch mehr vertonte Vorlesungen geben, statt manchmal nur die PDF Folien. Alles in Allem bin ich aber sehr zufrieden.
- Ich würde mir mehr Interaktion wünschen. Im Zuge dessen fände ich es durchaus interessant, das begleitende Seminar zur Neuro-VL als Videokonferenz durchzuführen
- Ich finde die Organisation wie sie Frau Vorwerk handhabt sehr gut. Das Lernmaterial in Form von kurzen Lernvideos zu den einzelnen Unterthemen finde ich wirklich äußerst ansprechend, kompakt und verständlich. Die Überprüfungsfragen bei kmed sind gut zur eigenständigen Überprüfung
- Mehr Schrifttext: ich habe gerade bei englischen Wörtern oder Fremdwörtern oft Probleme zu wissen, wie die genau geschrieben werden, wenn ich sie nur in einem Video höre, aber nicht geschrieben sehe. Für meine eigenen Lernzusammenfassungen muss ich diese Wörter dann oft erst googlen, bis ich das Richtige finde (als Beispiel: "Wolfram" basiert in Bezug auf das Target beim Thema "Aufbau und Funktionsweise eines Linearbeschleunigers")
- zu viele Leute für ein kleines Kurzthema eingeteilt (dies macht die Unterteilung innerhalb einer Gruppe schwierig)
- Die digitale Lehre von Fr. Prof. Vorwerk ist wirklich vorbildlich, ich würde nichts daran verändern.
- Ich finde die aktuelle Form der digitalen Lehre von Fr. Prof. Vorwerk in Form von Youtubevideos und bereitgestellten Übungsaufgaben in kmed absolut perfekt um zu lernen. Einzig eine einzelne Präsenzveranstaltung für einen Rundgang des Klinikbereichs und zum anschauen der Maskenherstellung etc. könnte ich mir als sinnvoll vorstellen.
- Die YoutubeVideos sollten beibehalten werden (falls das nicht sowieso geplant ist). Ich fand die Videos super gemacht und erklärt! Alles wurde gleich viel anschaulicher und leichter und schneller verständlich, als wenn man es nur erzählt bekommt/liest.
- Zu erwägen wäre, häufiger komplettere Inhalte/Folien weiterhin online zu stellen auch mit Audiodatei, da so adäquat für Prüfungen gelernt werden kann (besonders, wenn das Mitschreiben während einer Vorlesung schwer fällt)
- vertonte Online Vorlesungen und hochgeladene Lernmaterialien.
- Lehrvideos in denen man alles viel besser erkennt, als wenn jemand ganz vorne in einem riesen Raum etwas demonstriert und nur die erste Reihe es sehen kann.
- Meinetwegen könnte die Strahlentherapie immer so unterrichtet werden.
- Die Lernvideos und das digitale Lehrbuch sind perfekt für die Klausurvorbereitung und sollten beibehalten werden.
- Der YouTube Kanal mit den von Prof. Vorwerk hochgeladenen Videos ist super hilfreich und deckt die Grundlagen der Strahlentherapie ab. Darüber hinaus bietet es Möglichkeiten sich in bestimmten Themengebieten noch weiterzubilden, wenn entsprechendes Interesse vorhanden ist. Auch die kmed-Tests zur Überprüfung der gelernten Inhalte geben ein gutes Feedback darüber, ob man das zuvor Gelernte verstanden hat.
- Online Videos statt Seminare in Querschnittsfächern.
- Videos sollten auf YouTube erhalten bleiben. Sie sind gut und sehr verständlich aufgebaut. :)
- Gerne alles, es ist wirklich angenehm, mit den Videos und den dazugehörigen Kernfragen zu lernen.
- Online-Lernmaterialien, v.a. in Form von Lernvideos! (--> Prof. Vorwerks Lernvideos sind sehr gelungen und eignen sich hervorragend zum Lernen der theoretischen und zum Teil auch der praktischen Inhalte, jedoch sollte zumindest ein kleiner Teil in Präsenzform stattfinden, sodass man all das Erklärte auch einmal in Realität sehen und anwenden kann).
- Die Videos sind wirklich gut und können auch eine sinnvolle Ergänzung bieten, wenn wieder Präsenzunterricht stattfinden kann. Auch die Lernzielfragen haben mir gut geholfen bei der Rekapitulation des Stoffs.
- Es sollten die Videos mit den dazugehörigen Tests beibehalten werde
- Ich fand den Aufbau des Seminars sehr ansprechend (vom Fallbeispiel zur Diagnose und zur Therapie). Man wurde zum Denken angeregt und motiviert und es hat Spaß gemacht die Fälle zu bearbeiten! An einigen Stellen hätte ich mir jedoch genauere Erklärungen gewünscht (insbesondere von verschiedenen Abkürzungen wie TM, KM...).
- Sehr viel Einsatz der Professorin, schnelle und gute Antworten auf Fragen per E-Mail oder im Forum
- Die Videos von Frau Professor Vorwerk sind so gut, dass sie das Prinzip des Inverted Classroom perfekt möglich machen würden. Im Anschluss an das individuelle Lernen mit den Videos könnte man noch einige Seminare oder sogar Praktika veranstalten, um das gelernte Fallnahanzuwenden.
- Frau Dr Vorwerk hat wirklich grandiose Arbeit geleistet um ihren Teilbereich unseres Faches QB11_2 in digitaler Form anzubieten. Es ist die einzige Veranstaltung, die so in Präsenzlehre nicht besser laufen könnte. Ihre Videos sind perfekt mit dem KMED Lernkursverknüpft und in den Fragen prüft sie das wesentliche ab. Für uns Studenten war sie bisher auch sehr gut erreichbar. Daran können sich alle ein Beispiel nehmen. Wirklich super gemacht, Frau Dr. Vorwerk! Schade, dass für uns nur ein so kleiner Teil relevant ist.

# Free text evaluation of the pre-clinical questionnaire

- Abwechslungsreiches Format, "mal was neues", kann aber aus meiner Sicht keine live Veranstaltung ersetzen. Die kurzen Videos waren wichtig zur Auflockerung. Der Vorteil der "interaktiven Bilder" hat sich mir nicht erschlossen. Es war eher verwirrend, dass beim Bewegen des Cursors Textfelder aufgetaucht sind, die das Bild verdeckt haben. Abkürzungen in den Bildern (CTV etc.) sollten darunter nochmal erklärt werden. Technisch nicht optimal waren diese Balken zum draufklicken, wodurch z. B. MRT-Bilder ein- und ausgeblendet wurden, da man nicht genau erkennen konnte auf welchem Balken sich das Bild bezieht und relativ viel gescrollt werden musste
- Sehr geehrte Frau Professorin Vorwerk, dieses von Ihnen erstellte Lernmodul ist wirklich sehr ansprechend und interaktiv gestaltet worden.
- Besonders gut fand ich, dass man das Seminar in seinem eigenen Tempo durchführen konnte und das Seminar auch ggf. für eine kleine Pause pausieren konnte.
- Die interaktiven Bilder fand ich sehr gut.
- Sehr spannendes und interessantes Modul, gerne mehr davon in dieser Form, vielen Dank!!
- Ich fand das Programm sehr interessant. Die Videos haben einen guten Einblick in das Untersuchungs- & Therapieverfahren gezeigt
- Übungsaufgaben und Videos zu den Kursen hilfreich. Evtl. Videokonferenzen, um Wissenstand abzugleichen
- Man erkennt, dass Sie sich sehr viel Mühe gegeben haben, vielen Dank dafür.

# Free text evaluation of the clinical questionnaire

- großes Lob, so sollte digitale Lehre sein.kann für viele andere Kliniken ein gutes Beispiel sein
- Vielen Dank für die Mühe und die gute Lehre!
- Vielen Dank für die gute Umsetzung der Digitalisisierung! Es freut einen als Studenten wirklich, wenn Kliniken so viel Mühe in die Vermittlung von Unterrichtsstoff für ihre Studierenden stecken.
  Vielleicht könnten Sie einigen Klinken Nachhilfeunterricht geben. Die Kollgegen von der Urologie wären sicher dankbar... Die Studenten wären es auf jeden Fall.
- Starkes Konzept!
- leider waren die E-books nicht an allen Geräten nutzbar, das wäre ein Verbesserungsvorschlag. Ansonsten vielen Dank für Ihre Mühe und die tolle Lehre unter erschwerten Umständen! :-)
- insgesamt eine gute Vermittlung des Stoffes und sehr angenehm zum Zuhören. Sie haben sich viel mehr Mühe gegeben als die meisten anderen Dozenten
- Gute Idee mit den Youtubevideos. Kleine Zusammenfassungen zwischendurch wären für den Überblick hilfreich. Oder Fragen stellen, um das Wissen zwischendurch zu rekapitulieren.
- Vielen Dank für die Mühe, die Sie sich gemacht habe. Wirklich sehr vorbildlich und die Inhalte wurden sehr gut und lehrreich präsentiert.
- Im Gegensatz zu vielen anderen Fachdisziplinen war Ihre Abteilung überaus bemüht weiterhin gute Lehre zu machen. Der Aufwand der dafür ihrerseits betrieben wurde, ist bei den Studenten allgemein wahrgenommen und sehr gewertschätzt worden.
- Liebe Frau Prof. Vorwerk, vielen Dank für die tolle Online-Lehre! Das war echt eine super Lehre.
  Man konnte sich die Videos immer wieder ansehen und so viel mehr lernen als im Präsenzunterricht. Machen Sie weiter so!
- Vielen Dank für die Mühe, insgesamt sehr verständlich und ein tolles Angebot, die Tafelbilder könnten aber etwas detaillierter sein. :)
- Es war schwierig zu unterscheiden, welche Lerninhalte sich auf das 1.klinische Jahr und welche sich auf das 3.klinische Jahr beziehen. Eine eindeutigere Kennzeichnung und Zuordnung der Lerninhalte wäre hilfreich gewesen.
- Vielen Dank für Ihre Mühe!
- TOP Lehre dieses Semester, volle Anerkennung!!!
- Hallo Prof. Vorwerk, ich fand Ihre Umsetzung wirklich sehr gut! Vielen Dank für die Mühen. Vielleicht wäre eine Idee, nach bestimmten Abschnitten der Videos die Lernzielüberprüfungen auf kmed "anzukündigen". So nach dem Motto: "Bevor sie weiter machen, können sie zur Reflexion folgende Fragen beantworten...". So hatte das Dr. Schu in seiner VL gemacht. Natürlich können die Studierenden das auch von sich aus frei entscheiden. Vielen Dank noch mal und Liebe Grüße
- Bei manchen Videos wurde das Titelbild am Ende als Standbild angezeigt (Folie, die das Tafelbild wiedergegeben hat) bei anderen nicht. Vielleicht wäre es möglich diese Folien alle in einem PDF auf Kmed zu erhalten als Gedankenstütze.
- Ich möchte mich besonders für Ihre gut dargestellte und strukturierte Lehre auf Youtube bedanken. So müsste die gesamte Uni in dieser Coronazeit die Lehre machen. Bis dahin ist dieser Kurs ein positives "Unikat"
- Ich fand es schön, dass es Videos statt gesprochenen Präsentationen gab, da man mal ein nettes Gesicht dazu sah! Sie und die Strahlentherapie waren mit das einizige Fach, das sich solche Mühe gegeben hat, vielen Dank dafür!
- Eine bessere Strukturierung, um unsere Lerninhalte von denen des ersten klinischen Jahres abzugrenzen, wäre ideal; so, wie es momentan ist, musste man sich die Infos irgendwie zusammensuchen. Abgesehen davon fand ich die Mühe, die Sie sich mit den Videos gemacht haben, wirklich bemerkenswert. Das Resultat war auf jeden Fall sehr gut zum Lernen. Sehr schade finde ich, dass man nach diesem Semester vermutlich keinen Zugriff mehr darauf haben wird (abgesehen von den YouTube-Videos) - wäre es irgendwie möglich, diesen Lernkurs so freizuschalten, dass sich Studis aller Semester eintragen und weiterhin von Ihrem Engagement profitieren können? Vielen Dank jedenfalls, dass Sie sich die ganze Arbeit gemacht haben!
- Meiner Meinung nach haben Sie die Umsetzung der Lehre in ein digitales Format sehr gut bewerkstelligt. Die Art der Inhaltsvermittlung war sehr anschaulich und eingängig.
- Frau Prof. Dr. Vorwerk hat überragende Arbeit geleistet! Sie hätte die Videos nicht anschaulicher gestalten, nicht präziser formulieren können. Das Konzept auf Youtube wurde super umgesetzt. Es war ganz toll, dass es dazu noch einen Kmed Lernkurs gab, bei dem man Fragen beantworten konnte. Diese Fragen haben mir schnell gezeigt, auf welche Bereiche beim Lernen ich wert legen soll. Dass zu jeder Frage dann auch noch das passende Video verlinkt war, war einmalige Spitze. Frau Vorwerk hat auch sehr schnell auf eine Frage von mir geantwortet. Das ist leider nicht selbstverständlich in diesem Semester. Ich glaube, hier wurde das digitale Semester am Besten umgesetzt. Und man hat auch gemerkt, wie viele Mühe in diese Digitalisierung von ihr gesteckt wurde. Ganz großes Lob an Sie!
- Vielen Dank!
- Vielen Dank für dieses lehrreiche Lernangebot. Vor allem im Vergleich zu anderen Fächern, stachen Ihre Youtube Videos heraus. Die Tafelbilder waren sehr übersichtlich und auch die kurze Dauer der YouTube-Videos regten zum Lernen an.
- Die YouTube Videos waren richtig gut. Besonders gefallen hat mir, dass das Wissen in "Häppchen" vermittelt wurde, man also ganz gezielt einzelne Unterthemen nochmal nachschauen konnte ohne erst danach suchen zu müssen. Die Tafelbilder waren übersichtlich. Danke für die viele Arbeit, es hat sich gelohnt!
- Toll! Vielen Dank für das Engagement, Bitte auch anderen Kollegen vermitteln, dass das die Zukunft des Lernens ist!
- Die Kürze bei erhaltener Prägnanz der Videos hilft vor allem beim längeren Lernen nicht die Motivation zu verlieren.
- Die Videos waren anschaulich gestaltet und die Inhalte gut erklärt. Auch die Kürze der Videos war ideal, so konnte man ggf. nochmal ein Thema gezielt und effektiv wiederholen. Ebenfalls sehr gut gefallen haben mir die Übersichtsbilder mit den wichtigsten Fakten sowie die Kernfragen und die Probeklausur auf kmed. Dadurch konnte ich mein Verständnis für die Lerninhalte prüfen. Alles in allem war die Veranstaltung Strahlentherapie mit Abstand die beste im "digitalen" Semester!
- Die Kurzvideos sind super! Man kann sich den Stoff so gut einteilen und immer wieder anschauen.
- Ich finde, dass Frau Professorin Vorwerk eine vorbildliche Lehre betreibt, die den Studenten sehr motiviert.
- Die mit Abstand bester Lehrveranstaltung in diesem Semester haben die Youtube-Videos zum Prostata-Karzinom dargestellt. Wenn alle Inhalte so gut vermittelt werden würden wäre das Studium sehr viel besser.
- Man könnte final eine PP Präsentation erstellen mit allen Tafelbildern kumuliert, damit man sich noch einmal durch visuelle Wdh. an das Wichtigste erinnern kann.
  Trotzdem ist das Format jetzt schon wirklich toll. Daran können sich viele Bereiche ein Beispiel nehmen (Gerne mal den Urologen weitergeben, die ihre Folien noch nicht mal besprochen haben). Vielen Dank für Ihre Mühe, ich konnte dadurch sehr viel mitnehmen!
- Ein wirklich großes Lob an die Digitalisierung der Lerninhalte in Form der Youtube Videos. Die wichtigsten Informationen sind dort in präziser und angemessener Weise hervorragend zusammengefasst. Meiner Meinung nach ist dadurch das Lernen im "Coronasemester" im Vergleich zu vielen anderen Fächern mit Abstand am besten umgesetzt worden.
- Die kmed Kurse waren sehr übersichtlich und gut verständlich!
- Vielen, vielen Dank für die großartigen Vorträge auf Youtube!!! Verglichen mit den anderswo hochgeladenen Power Point Präsentationen waren diese deutlich lehrreicher. Abgesehen davon, haben mir die Präsentationen den Bereich der Strahlentherapie noch einmal deutlich näher gebracht! Auch für die Zukunft ist es toll diese Videos für einen Überblick zur Hand zu haben.
- -vielen Dankt für die einzig sinnvolle Lehre dieses Semester.
- Ein bis zwei Folienbilder mehr zur schnellen Auffrischung, nach dem man die Videos gesehen (z.B. im Ebook) wären super. Die Videos sind sehr hochwertig, ein mehrfaches Ansehen aber teilweise zu umständlich im Vergleich zu einfachen Bildfolien. Insgesamt war der digitale Unterricht zur Strahlentherapie aber um 2-3 Ligen besser, als es noch die Seminare im damaligen 5. Semester waren. Ein weiterer Ausbau in diese Richtung wäre daher super!
- Die Videos waren wirklich klasse und eine sehr hilfreiche Unterstützung beim Lernen. Der einzige kleine Verbessungsvorschlag wäre: weniger kleine Videos, sondern zusammengeschnitten
- Um den Unterricht zu ersetzen eignen sich die Videos auf Youtube besser als reine Power point Präsentationen und sich auch angenehmer anzusehen.
  Für die Vorbereitung auf die Klausur wäre es allerdings nett die Tafelbilder/Folien die genutzt wurden noch einmal in digitaler Form zum durchklicken zu haben. Da sich das anschauen in den Videos doch als deutlich aufwendiger erwiesen hat.
- Ich fand die angebotenen Videos sehr gut. Ich hätte mir nur von den anderen Fächern mehr (als nichts) Angebote für das digitale Lernen gewünscht. Vielen Dank und viele Grüße
- Sie haben sich wirklich sehr viel Mühe gegeben und waren jederzeit für uns ansprechbar! Vielen Dank für Ihre Unterstützung!
- Die Lernvideos sind sehr gut gestaltet und definitiv sehr hilfreich.
- Ich finde die Tafelbilder gut, weil Sie da mit der Hand auch auf Schemata oder Strukturen zeigen können, über die Sie gerade sprechen. Außerdem kann man anhand der Überschriften der Kurzvideos abschätzen, ob das folgende Video in dem Moment relevant für das eigene Interesse ist. Die Darstellung mit den Links in den Unterordnern auf kmed ist noch etwas verzerrt, aber sicher leicht zu lösen. Bei den Kernfragen wären Lösungen am Ende hilfreich. Die Vorträge halten Sie sehr strukturiert und souverän. Weiter so!
- Vielen Dank für die tollen Videos! Kombiniert mit Kleingruppenseminaren halte ich diese Art der Lehre für sehr gut, um die manchmal komplexen Themen der Radiologie zu verstehen.
- Die Lernvideos auf youtube waren sehr gut. Auch welche Themen für uns und welche für das andere Semester bestimmt waren.
- Es wäre schön, wenn die Lernziele deutlicher gezeigt wären und kleine Lernzielüberprüfungen miteingebaut wären.
- Ich fand die Videos und die Tafelbilder wirklich sehr gut und hilfreich. Die Strahlentherapie war das einzige Fach von diesem Kurs, das sich Mühe bei der Digitalisierung der Lehre gegeben hat. Und wo es auch mal klare Ansagen zur Klausur, etc. gab.
- Vielen Dank für die sehr lehrreichen Kurzvideos auf Youtube, die wirklich gut strukturiert waren und das Lernen sehr erleichtert haben! Besonders gut finde ich, dass man die Kapitel einzeln auswählen kann, sodass bei Wissenslücken ein effektives Nachschlagen möglich ist.
- Liebe Frau Vorwerk, Vielen herzlichen Dank für Ihr Engagement und Ihr klug durchdachtes Kopnzept. Die YouTube Videos waren sehr gut strukturiert und klar verständlich formuliert. Ich denke, sowohl für uns Studierende als auch für Patienten. Die Tafelbilder waren hilfreich, jedoch zum späteren Wiederholen nur erreichbar (vertu ich mich?) durch erneutes Abspielen der Videos. Vielleicht wäre hier auch erneut ein kleines Skript oder eine Datei mit den Screenshots dieser Tafelbilder sinnvoll. Der unkomplizierte Kontakt an die Studierenden via Email oder Kmed Forum war auch super. Wir können uns nur wünschen, dass andere Dozenten sich an Ihnen ein Beispiel nehmen. Auch in der Curriculumskommission ist aufgefallen, wie sehr Ihnen gute Lehre am Herzen liegt. Das ist (leider) heute nicht mehr selbstverständlich. DANKE dafür.
- Frau Prof. Vorwerk hat gezeigt, wie man mit viel Mühe und Liebe zum Detail digitale Lehre gestalten kann. Die Kombination zwischen youtube-Kanal und Ebook ist perfekt, da sie gut aufeinander abgestimmt sind. Der youtube-kanal ist übersichtlich aufgebaut und mit den Playlists kann man gezielt das lernen, was man gerade braucht. Frau Prof. Vorwerk schafft es, in 2-5 minütigen Videos alles wichtige auf den Punkt zu bringen und so die Lernmenge in kleine Häppchen zu verpacken. Im Vergleich zu besprochenen Vorlesungen, kann man dabei besser mal Themen überspringen, wiederholen oder in schnellerer Geschwindigkeit anhören. Bei 1,5h VL verliert man da schnell den Überblick. Im Vergleich zu anderen kmed Lernkursen ist auch dieser Übersichtlicher und Interaktiver, außerdem ist direkt unter den Fragen verlinkt, welches Video man bei Falschbeantwortung nochmal ansehen sollte. Durch das Forum bestand immer Kontakt zur Dozentin wenn doch mal fragen waren. Ich finde es auch sinnvoll, dass das Format des youtube-Kanals ja so nicht nur für Studierende zugänglich ist sondern auch für Auszubildende, Pat. und interessierte Laien. Einziger Tipp für die Tafelbilder: Die handschriftlichen Tafelbilder kann man zwar auch sehr gut lesen und sie sind etwas flexibler zu gestalten, allerdings sind die PC-generierten auch sehr schön und wenn diese sowieso erstellt werden, kann man sich vielleicht die Arbeit sparen, alles nochmal an die Tafel zu schreiben. Das ist also nur eine pragmatische Anmerkung. Insgesamt: 1+ mit Sternchen!
- Die Playlist auf Youtube fande ich sehr gut. Ergänzen könnte man dies durch ein Skript aus den Tafelbildern. Die einzelnen Videos waren sehr gut erklärt und die Tafelbilder sehr übersichtilich. Die Kmed-Lernkurse empfinde ich als lästig. Besser wäre ein Skript anstelle des kmed-Lernkurses. Außerdem habe ich die Anweisungen für dieses Semester als sehr unübersichtlich empfunden, da sie mit den Anweisungen des 5. Semesters zusammen waren.
- Die Videos waren sehr hilfreich und verständlich. Ich fand es gut, dass es bei den verschiedenen Erkrankungen nicht nur um Strahlentherapie ginge, sondern auch um andere Fachrichtungsdisizplin. Die Art und weise war sehr gut, ich hoffe, dass andere Dozenten sowas machen. ich bin sehr begeistert davon.
- Ich finde die Digitalisierung der Lehrmaterialien war gut durchdacht und Prof. Dr. Vorwerk hat sich wirklich viel Mühe gemacht uns den Lehrinhalt nahe zu bringen. Für die Zukunft ist die einzige Verbesserung die mir einfällt eine Möglichkeit des interaktiveren Austausches z.B. mittels Zoom-Seminar, wo Fragen gestellt werden können.
- Klasse, das Sie sich so viel Mühe gegebenen haben, um ein digitales Lehrkonzept zu entwickeln, das den Präsenzunterricht einigermaßen ersetzt. Vielleicht können Sie das einigen anderen Fachbereichen vorstellen in der Hoffnung, dass diese sich etwas Vergleichbares überlegen...
- Sehr geehrte Frsu Vorwerk, Vielen herzlichen Dank für Ihre Mühe, ein digitales Lernangebot für die Studenten trotz der turbulenten Coronazeit einzurichten. Es ist Ihnen HERVORRAGEND gelungen!!
  - Der Youtube-Kanal ist sehr übersichtlich gestaltet
  - Die Videos sind auf den Punkt gebracht, die Tafelbilder übersichtlich und ansprechend gestaltet und gut geeignet zum Lernen
  - Auf kmed ist der Bezug zu den Playlists auf Youtube klar und nachvollziehbar
  - Die Kernfragen auf kmed sind gut geeignet zur Wissensüberprüfung
  - Die Tafelbilder auf kmed hochzuladen, bietet den Studenten eine gute Alternative, abseits der Youtube-Videos zu lernen und das Wissen zu wiederholen
  Ich habe viel Verständnis dafür, dass in einer Ausnahmezeit wie dieser coronabedingten die Lehre hier und da zu kurz kommt. Leider haben andere Fachrichtungen aber gar nicht mit uns kommuniziert oder neue digitale Inhalte zur Verfügung gestellt. Angesichts dieser Tatsache rechne ich es Ihnen hoch an, dass Sie viel Arbeit und Zeit in unsere Lehre gesteckt haben und auch die Kommunikation sehr klar war. Sie haben es sogar geschafft, mein Interesse am Fach Radiologie, welches vorher nicht sehr ausgeprägt war, zu wecken! Herzlichen Dank! So macht Lehre Spaß!
- Folien, Übungen und digitale Seminare sollten möglichst kompakt sein. Wenn jede Fachrichtung 80 PP-Präs., 20 Seminare etc. in genauso vielen Unterordnern hochlädt, dann verwirrt man nur! Deshalb ist es einfacher morgens früh aufzustehen und zum Seminar zu fahren, als bei K-MED durchzusehen. VG
- Hallo Frau Vorwerk, erst einmal vielen Dank für die Videos. Die waren wirklich sehr gut für die Vorbereitung. Das Format hat mir persönlich sehr gut gefallen, da man bei Dingen, die man genauer Wissen wollte zurück spulen konnte und sie sich noch einmal anhören konnte. Auch die Tafelbilder waren wirklich gut. Schade natürlich dass der interaktive Unterricht nicht stattfinden konnte. Natürlich beantwortet Google auch die meisten Fragen aber meistens merkt man es sich besser, wenn man es im Unterricht erarbeitet hat. Zu der Struktur auf k-med. Leider fand ich die Anordnung der Ordner etwas unübersichtlich und habe mir schwer getan, durchzublicken, was ich schon angesehen hatte und was nicht. Sehr gute Lehre von Ihnen. Vielen Dank.
- Die Videos zum Prostatakarzinom waren sehr gut gemacht und extrem hilfreich beim Lernen. Dankeschön :)
- Super Videos mit tollem Engagement von Prof. Vorwerk!
- Mir haben die themenbezogenen Videos gut gefallen, v.a. dass die Videos kurz und in einzelne Kapitel untergliedert waren. Habe dank der Videos einen guten, sehr strukturierten Eindruck erhalten vom Prostatakarzinom und habe sogar mehr mitnehmen können als während eines Seminars.
